# Graph Neural Network and LSTM Integration for Enhanced Multi-Label Style Classification of Piano Sonatas

**DOI:** 10.3390/s25030666

**Published:** 2025-01-23

**Authors:** Sibo Zhang, Yang Liu, Mengjie Zhou

**Affiliations:** 1 School of Arts, Shandong University, Jinan 250100, China; sibozhang_sdu@ieee.org; 2 Department of Computer Science, Worcester Polytechnic Institute, Worcester, MA 01609, USA; harryliu@ieee.org; 3 Department of Computer Science, University of Bristol, Bristol BS8 1QU, UK

**Keywords:** music analysis, piano sonata analysis, big data, neural networks

## Abstract

In the field of musicology, the automatic style classification of compositions such as piano sonatas presents significant challenges because of their intricate structural and temporal characteristics. Traditional approaches often fail to capture the nuanced relationships inherent in musical works. This paper addresses the limitations of traditional neural networks in piano sonata style classification and feature extraction by proposing a novel integration of graph convolutional neural networks (GCNs), graph attention networks (GATs), and Long Short-Term Memory (LSTM) networks to conduct the automatic multi-label classification of piano sonatas. Specifically, the method combines the graph convolution operations of GCNs, the attention mechanism of GATs, and the gating mechanism of LSTMs to perform the graph structure representation, feature extraction, allocation weighting, and coding of time-dependent features of music data layer by layer. The aim is to optimize the representation of the structural and temporal features of musical elements, as well as the dependence between discovery features, so as to improve classification performance. In addition, we utilize MIDI files of several piano sonatas to construct a dataset, spanning the 17th to the 19th centuries (i.e., the late Baroque, Classical, and Romantic periods). The experimental results demonstrate that the proposed method effectively improves the accuracy of style classification by 15% over baseline schemes.

## 1. Introduction

From the 17th to the 19th centuries, the piano sonata played a pivotal role in shaping the musical landscape in Europe while undergoing significant transformations that reflected advances in both musical aesthetics and piano construction. This evolution of piano sonata, including forms, performing techniques, and depth of expression, was largely driven by eminent composers such as D. Scarlatti, Haydn, Mozart, Beethoven, and Schubert. In this study, we analyze piano sonatas as complete multi-movement works rather than focusing solely on sonata form movements. A piano sonata typically consists of three to four movements, with the first movement usually following sonata form. Sonata form refers to a specific musical structure with exposition, development, and recapitulation sections, while ‘sonata’ more broadly describes a multi-movement work for piano. These composers not only enriched the repertoire by writing a plethora of piano sonatas but also pioneered innovations that continue to influence music theory and practice. Particularly notable is Beethoven’s anthology of 32 piano sonatas, often regarded as the “New Testament” of piano music, which represents a pinnacle in terms of structural, harmonic, and melodic mastery.

Traditional musicological studies usually describe the development of the piano sonata in terms of harmony, thematic construction, and the sonata form, but it is often difficult to dissect the internal complexities of various musical styles and the deeper connections among compositional styles [1,2,3,4,5,6]. The advent of big data and advances in computing technologies [7,8,9,10] have created opportunities for the in-depth and comprehensive evaluation of musical compositions. In this study, GNNs (GCNs and GATs) and LSTMs are combined to capture the structural (chords, pitch, and duration) features and temporal (rhythmic patterns and melodic directions) features of the data. In this way, the style of piano sonatas can be classified accurately, and the historical depth of the genre can be explored and reproduced.

The style classification of piano sonata includes collecting MIDI files, data preprocessing, feature extraction and learning, and multi-label prediction. We propose a model integrating GCN, GAT, and LSTM to complete the classification. We have designed algorithms and experiments to improve the performance of our model. The approach facilitates a more detailed classification of piano sonata styles, which can lead to a deeper understanding of the collaborative inheritance relationships among composers, and the sentiment values for sonata works.

The contributions of this research include the following.

- We propose a novel integrated approach using GCNs, GATs, and LSTMs to analyze piano sonatas and provide a new perspective on music classification. To the best of our knowledge, this study proposes the first GNNs-based analysis of piano sonatas.
- The experimental results show that the trained model significantly improves the accuracy of style classification compared with the baseline schemes and offers new insights into the deep pattern in musical structures.
- We construct an open dataset of piano sonata MIDI files (https://github.com/SybilZhang123/Piano_Sonata_17_19c_MIDI_File (accessed on 20 March 2024): An open resource of MIDI files of famous piano sonatas composed in the 17th–19th century) that serves as a new resource for subsequent music analysis research, thereby enriching the field with detailed data on historical music compositions.

The rest of the paper is organized as follows. Section 2 presents a review of related work in the field of music analysis and machine learning. Section 3 describes the application of GNNs and LSTMs in music research. Section 4 describes the system model and the optimization targets. Section 5 details the design and implementation of the algorithm. In Section 6 and Section 7, we present the experiments and the conclusions, and discuss avenues for future research.

## 2. Related Work

In this section, we summarize the important results referenced in the paper, including works on deep learning, music classification, and multi-label automatic classification.

### 2.1. Deep Learning for MIDI Format Data

Deep learning methods are widely used on data in MIDI format, especially for the generation and classification of music. For example, Huang et al. [11] used LSTMs-based methods to learn the structure and features of music from MIDI files and generate new musical compositions with chords and melodies. Tang et al. [12] performed preprocessing operations such as track separation, musical feature extraction, and data vectorization on MIDI files based on LSTMs to identify musical styles and generate music. These methods involving data in MIDI format have been used for performance teaching and music theory [12,13].

### 2.2. Integration of Deep Learning in Music Analysis

Recent advancements in deep learning have significantly enhanced music analysis, particularly through the application of GNNs and LSTMs. GNNs are adept at modeling complex dependencies in music compositions, such as those among notes, chords, and melodies, owing to their capacity to handle graph-structured data [14,15,16,17,18,19]. Conversely, LSTMs excel in capturing the temporal dynamics of music and are thus ideal for analyzing themes and variations within sonatas [20,21]. The synergistic integration of these technologies has opened novel pathways for both style classification and emotional analysis of piano sonatas, marking a advance in computational musicology.

### 2.3. Innovations in Music Style Classification

In the realm of music classification, Sun et al. [22] introduced a hybrid model that integrates CNNs with GNNs to analyze the time–frequency features of and structural information in audio signals. Their methodology, which optimizes edge weights in a Siamese network configuration, has not only enhanced the accuracy of music style classification but also underscored the utility of GNNs in dissecting complex musical structures. Their study provides a basis for the application of similar approaches to the style classification of piano sonata.

### 2.4. Functional Harmonic Analysis Using GNNs

Huang et al. [23] explored the use of GNNs for chord function analysis, an approach that leverages the interrelations among notes to extract functional harmonic information. This novel analytical tool is useful for deconstructing the harmonic frameworks prevalent in classical and romantic music and offering fresh insights into the underlying structures of sonatas [24].

### 2.5. LSTMs and Long-Term Dependency Modeling in Music

LSTMs have been extensively applied to model long-term dependencies in musical sequences, thereby facilitating the generation of complex polyphonic music. This research has not only advanced the understanding of melodic and harmonic constructions in music but also laid the groundwork for employing LSTMs in the nuanced analysis of piano sonatas concerning both style and emotional content [25].

In summary, the application of GNNs and LSTMs represents a transformative approach in the field of music analysis. These methods have enriched musicology research and provided a valuable framework for applications in music education and recommender systems [11,12,13,26,27]. This study explores the use of these advanced deep learning tools to assess the styles of piano sonatas composed in the 17th to 19th centuries and identify an efficient method for the style classification so as to provide a basis for discovering deeper connections among the sonata works, and addresses the limitations of traditional musicological studies.

## 3. Background

In this section, we introduce the theoretical background of GCNs, GATs, and LSTMs and explore their application to sonata analysis and their potential value for piano sonata style classification.

### 3.1. Neural Networks and Music Analysis

The field of music analysis based on neural networks includes music feature extraction and representation learning, music classification and label prediction, and music generation and music information retrieval. Our research deals specifically with feature extraction and representation as well as music classification and label prediction. The structural and temporal features of music are used in the classification of piano sonata, especially note characteristics (pitch, duration, loudness, etc.), harmony, modal, tonality, melodic direction, and rhythmic patterns. Feature representation serves to convert these high-level musical features into more structured and interpretable feature vectors that provide key inputs for the delineation of the stylistic features of piano sonatas. Based on the advantages of GCNs [28], GATs [16], and LSTMs in mining structural and temporal features, we choose them for use in the classification.

### 3.2. Structural Characteristics of GNNs and Piano Sonatas

GNNs are based on graph theory and deep learning and, unlike CNNs, extend the concept of deep learning to non-Euclidean structured data, a feature that is particularly suitable for revealing the intricate structures and relationships in musical compositions. For example, GCNs effectively represent the features of nodes (e.g., musical notes) through the adjacency matrix, while the attention mechanism enables GATs to assign levels of importance to various neighbours during the feature aggregation process, thus improving the performance of GCNs. Collaboration between the two GNNs is essential for identifying the key structural features (chords, pitch, and duration) of a sonata that define the composer’s style or identity. Based on the above analysis, we propose a model that integrates GCNs and GATs, and design algorithms to analyze sonata styles based on sonata structure feature map data.

### 3.3. Temporal Characteristics of Piano Sonatas and LSTMs

Because LSTMs utilize gating units and Long Short-Term Memory units as the core technology for controlling information flow, memory updating, and storage, they are adept at learning long-term dependencies and capturing patterns and dependencies in sequential data. For example, LSTMs can filter and preserve key temporal features (rhythmic patterns and melodic directions) in music data through the three gating units (the oblivion gate, the input gate, and the output gate) in ways that help reveal the inheritance and development of works and composers as well as the formal evolution of piano sonatas. Therefore, our model also integrates LSTMs as a component to capture temporal features in the music data and, thus, complement the GNNs (GCNs and GATs).

### 3.4. Piano Sonata Data Preprocessing and Encoding

In order to ensure the quality of the data, reduce the interference from inferior audio signals, and facilitate the processing of GNNs and LSTMs, we use MIDI format files for the preprocessing and encoding. The preprocessing and encoding consist of four main steps: extracting and parsing the notes from the MIDI files to capture the pitch, duration, and intensity; quantifying rhythms to standard musical beats such as quarter notes and eighth notes; performing a harmonic analysis to identify the predominant chords and key signatures (with each chord assigned a specific code, e.g., C major coded as “001” and G major as “002”); and constructing the graph of a sonata in which the nodes and edges are defined based on music theory, such as chord progressions, melodic connections, and rhythmic patterns.

### 3.5. Relation Mining and Style Classification

Different from the traditional musicological study of the stylistic evolution of piano sonatas, we perform the multi-labeling and classification based on the high-level features of the music data while overcoming the ambiguity of the stylistic boundaries and, ultimately, improving the accuracy of the classification. Specifically, we use the grasp of features such as notes and harmonies by GNNs to determine the labels for works such as “Classical” and “Baroque”, and we use the grasp of temporal features such as rhythms and melodies by LSTMs to determine the inheritance and development of works as well as the direct and indirect influences among composers (e.g., the motifs and themes in Beethoven’s early sonatas that resemble those in Haydn’s works). Therefore, the integration of GNNs and LSTMs helps to achieve the efficient stylistic classification of works based on existing musicological theories.

In conclusion, the integration of these methods is, then, intended to reveal the complex interrelationships in piano sonata works and provide new insights into style classification. We design an algorithm based on these tools, the details of which are discussed in Section 5.

Our work addresses stylistic ambiguity through a multi-faceted analysis combining structural and temporal features. The GNN components analyze harmonic progressions and melodic structures characteristic of each period, while the LSTM tracks temporal evolution of these features. For example, when analyzing works from transitional periods, such as late Beethoven sonatas that bridge Classical and Romantic styles, our model considers both Classical elements (formal structure) and Romantic features (harmonic innovation). The model assigns probability scores across multiple style categories rather than enforcing strict boundaries, allowing works to be classified along a stylistic continuum. This better reflects the gradual evolution of compositional styles and helps capture hybrid works that combine elements from multiple periods.

## 4. System Model

The following discussion elaborates on the multi-label classification task of piano sonatas from the 17th to 19th centuries and the relationships among musicians using neural networks. The model uses the heterogeneous integration of GCNs, GATs, and LSTMs to model and learn the structural and temporal features of music.

### 4.1. Overview of the Music Analysis Framework

**Figure 1:**
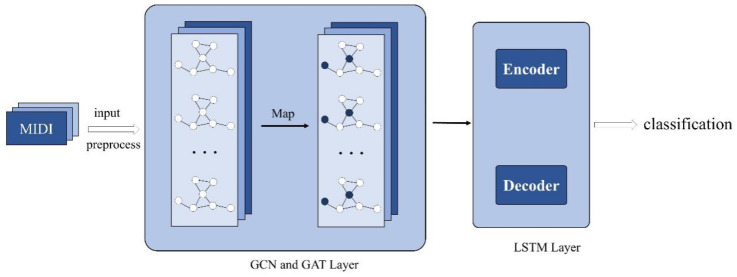
The structure of the proposed integrated model.

Our framework combines the advantages of GNNs and LSTMs for the learning and identification of the structural and temporal characteristics of piano sonatas from preprocessed graph data samples. The analysis involves describing the organization of music in the vertical dimension, such as pitch and harmony, as well as in the temporal dimension, such as rhythm structure and duration. Thus, the GNNs explain the correlations and differences among works by analyzing chord progression patterns and melodic changes. The LSTMs track changes in tempo and rhythmic patterns, revealing the hidden compositional and aural inertia behind the works while also exploring the subtle stylistic differences between the works and the inheritance and developmental relationships between and among composers. In this way, the combination of GNNs and LSTMs improve the classification efficiency of the style analysis in traditional musicology. The diagram of the model is shown in Figure 1, and the main notations used in the paper are summarized in Nomenclature.

### 4.2. Data Preprocessing and Graph Construction

Sonata data are preprocessed to identify them into attributes such as pitch and duration. Then, based on music theory, a graph structure composed of nodes and edges is constructed. The preprocessing of these data includes extracting the notes from the MIDI files, rhythm quantization, and harmonic analysis. Typically, the preprocessing consists of three steps.

- Note Extraction. The fundamental attributes of each note, including pitch, duration, intensity, and start time, are extracted from the MIDI files. These attributes provide the basic “node” data for constructing the music graph.
- Duration and Rhythm Quantization. The durations of the notes are standardized and aligned to the nearest musical beat, such as quarter notes or eighth notes, to simplify the rhythmic structure and facilitate the model’s recognition and processing of musical rhythms.
- Harmonic Analysis. Chord progressions are analyzed to determine the key and main chords of a work. Each chord and key note is assigned a unique identifier to facilitate its representation in the graph as relationships between chords and melodies.

Following the preprocessing steps, a graph structure is meticulously constructed from the nodes and edges based on music theory and the relationships derived from the preprocessed data. In the graph, each node represents a distinct musical element derived from the three preprocessing steps.

- Pitch nodes represent individual pitches extracted from the sonatas, reflecting the tonal aspects of the music.
- Rhythm nodes are derived from the quantized rhythmic data to capture the temporal patterns within the compositions.
- Harmony nodes are identified based on each chord and key from the harmonic analysis to encapsulate the harmonic framework of the sonatas.

On the other hand, the edges of the graph represent the relationships among the various musical elements.

- Melodic edges connect sequential pitch nodes within a melody line to indicate the progression of the pitches in a piece of music.
- Rhythmic edges link nodes that share rhythmic patterns, thus highlighting the rhythmic continuity and transitions within the sonata.
- Harmonic edges connect harmony nodes based on the chord progressions and key transitions identified during the harmonic analysis to illustrate the movement and modulation between harmonic states.

### 4.3. The Principles of Integrating the GCN, GAT, and LSTM

In the system model, the first key component is the GCN. In the analysis of the sonatas, the GCN learns the structure of musical works, including the harmonic progressions, melodic lines, and rhythmic patterns. The GCN employs an updating rule to aggregate information from neighboring nodes [27,29]:

(1) $$H^{\left(l+1\right)}=\sigma \left({\hat{D}}^{-1/2}\hat{A}{\hat{D}}^{-1/2}H^{\left(l\right)}W^{\left(l\right)}\right)$$

where $H^{\left(l\right)}$ is the representation of the first-layer nodes, $\hat{A}$ is the adjacency matrix with self-connections, $\hat{D}$ is the corresponding degree matrix, $W^{\left(l\right)}$ is the weight matrix of that layer, and σ is the activation function (e.g., ReLU). In music analysis, the nodes can represent notes or chords, and the edges represent the temporal or harmonic relationships among notes. The GCN model learns to combine these musical elements into complex structural patterns that serve to determine the style of music pieces. The GAT, the second key component, enhances the information aggregation process from the nodes by introducing an attention mechanism in addition to the GCN and improves the efficiency and accuracy of learning so that the model can assess the relative importance of each node. Updating GAT follows a set of rules [27,29]:

(2) $$Attention\left(e_{ij}\right)=\mathrm{LeakyReLU}\left(a^{T}\left[W^{\left(l\right)}h_{i}∥W^{\left(l\right)}h_{j}\right]\right)$$

(3) $$\alpha_{ij}=\frac{\exp\left(Attention(e_{ij})\right)}{\sum_{k\in N(i)}\exp\left(Attention(e_{ik})\right)}$$

(4) $$h_{i}^{\prime }=\sigma \left(\sum_{j\in N(i)}\alpha_{ij}W^{\left(l\right)}h_{j}\right)$$

where $h_{i}$ represents the feature vector of node *i*, $e_{ij}$ represents the edge between nodes *i* and *j*, $a^{T}$ is the parameter vector of the attention mechanism, $\alpha_{ij}$ represents the attention weights reflecting the importance of node *j* for node *i*, $W^{\left(l\right)}$ is the weight matrix, and σ is the activation function. GATs can identify the importance of various musical elements. For example, in a chord progression, GATs can highlight certain chords that may have greater significance than others by learning their relationships.

In the system model, the third key component is the LSTM, which is integrated to process the temporal features in the music data and complement the structural analysis provided by the GCN and the GAT. In music analysis, LSTMs can be used to identify and learn melodic developments, rhythmic changes, and dynamic fluctuations. For example, in a complex piano sonata, the LSTM can track the development of and transitions in the themes, even when the transitions are over long intervals.

The status update for each time step follows a set of rules [27,29]:

$$\begin{array}{cc} \mathrm{Input}\;\mathrm{gate}:\;i_{t} & =\sigma \left(W_{i}\cdot \left[h_{t-1},x_{t}\right]+b_{i}\right)\\ \mathrm{Oblivion}\;\mathrm{gate}:\;f_{t} & =\sigma \left(W_{f}\cdot \left[h_{t-1},x_{t}\right]+b_{f}\right)\\ \mathrm{Output}:\;o_{t} & =\sigma \left(W_{o}\cdot \left[h_{t-1},x_{t}\right]+b_{o}\right)\\ \mathrm{New}\;\mathrm{memory}\;\mathrm{unit}:\;g_{t} & =\tanh\left(W_{g}\cdot \left[h_{t-1},x_{t}\right]+b_{g}\right)\\ \mathrm{Updated}\;\mathrm{cell}\;\mathrm{state}:\;c_{t} & =f_{t}\cdot c_{t-1}+i_{t}\cdot g_{t}\\ \mathrm{Updated}\;\mathrm{hidden}\;\mathrm{state}:\;h_{t} & =o_{t}\cdot \tanh\left(c_{t}\right) \end{array}$$

The above parameter vectors, (W) and (b), are the parameters learned during training and determine how the LSTM updates its state given an input and the previous state.

In summary, after the initial stages of the model, then the GCN, with its adjacency matrix and node feature aggregation functions, is able to capture the direct connections among the musical elements. After the data flow to the GAT layer, the GAT, through its attention mechanism, further enhances the model’s recognition of important relationships within the music structure. After the GAT processes the structural features, its output is transformed into sequence data, which are inputted into the LSTM layer. Notably, the LSTM can process the time sequence data obtained from the GAT to uncover the long-term temporal dependencies and dynamic changes in music.

### 4.4. Loss Function Design and Optimization

The training of the model involves multiple steps that optimize the parameters of the GCN and GAT for learning and predicting the relevant stylistic and performative characteristics of musical pieces. During this process, the loss function typically used is the cross-entropy loss for classification tasks, optimized as

(5) $$L=-\sum_{i=1}^{N}\sum_{c=1}^{C}y_{ic}\log p_{ic}$$

where *N* is the number of samples, *C* is the number of classes, $y_{ic}$ represents the true label of node *i* (using one-hot encoding), and $p_{ic}$ represents the model’s predicted probability that node *i* belongs to class *c*. To optimize the model, we employ the stochastic gradient descent (SGD) algorithm and its advanced variant, the Adam algorithm. These optimization techniques are crucial for fine-tuning not only the parameters of the GCN and GAT but also the weights in the LSTM layer to ensure the overall effectiveness of the model in music analysis. The multi-label cross-entropy loss function enables the model to express stylistic ambiguity by allowing partial membership across categories. When processing transitional works, such as early Romantic sonatas that retain Classical features, the model can assign significant probabilities to both categories rather than forcing a binary classification.

## 5. Algorithm Design

This section details the motivations behind the algorithm design, the pseudocode, the algorithm description, and the formulation of the multi-label cross-entropy loss function.

### 5.1. Overview of the Proposed Algorithm

**Figure 2:**
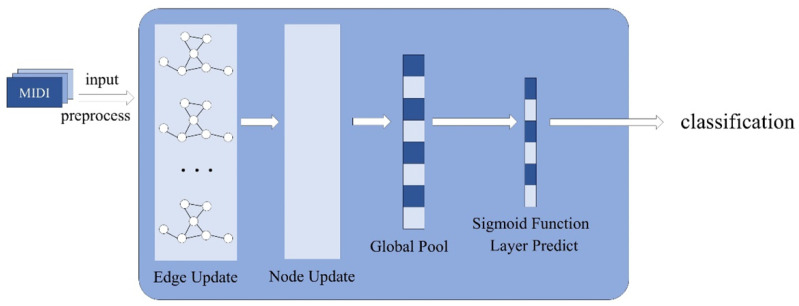
Detailed structure of the proposed GAT-LSTM algorithm.

Traditional LSTM methods excel in capturing temporal features but have difficulty processing graph-structured data. Conversely, traditional GNN methods are adept at handling structural features but lack the capability to capture temporal dynamics [21]. To overcome these limitations in processing complex musical data, we propose the GAT-LSTM algorithm, which efficiently integrates the processing of structural and temporal data by introducing GAT between GCN and LSTM as depicted in Figure 2. This integration enables the model to understand the intricate relationships within musical data comprehensively.

First, technically, the proposed GAT-LSTM algorithm integrates GCN, GAT, and LSTM. GNNs and LSTM preprocess and learn both structural features, such as pitch, duration, and chords, and temporal features, such as rhythm, beat, and melody, based on the musical information contained in MIDI files and following the logical order dictated by musicology. The GAT between the GCN and LSTM layers not only optimizes the learning efficiency of GCN through weighted attention but also aids LSTM in temporal modeling, thereby achieving precise feature learning. The result is a comprehensive and accurate understanding of the characteristics of piano sonatas.

Second, to ensure the superior performance of the model in music analysis tasks, we employ various advanced training strategies, including the design of appropriate loss functions, efficient optimization algorithms, and model validation. Third, to achieve accurate label output while considering the possibility of samples belonging to multiple categories, we utilize a softmax activation function to convert the output of the fully connected layer into a probability distribution so that the model produces probabilistic predictions for each category to facilitate multi-label classification tasks. In the following discussion, we explain the algorithm through pseudocode and other aspects. Notably, in the proposed algorithm, the GAT dynamically adjusts the adjacency relationships in the graph structure through an attention mechanism, thereby enhancing the feature representation capabilities of the GCN and providing more refined input features for the LSTM. By contrast, traditional GNN and LSTM methods, when used independently, cannot simultaneously handle structural and temporal features, resulting in suboptimal performance on complex data classification tasks. By positioning the GAT between the GCN and LSTM layers, the algorithm leverages the strengths of each component to achieve the more precise classification of musical data.

### 5.2. Algorithm Pseudocode

The algorithm pseudocode is shown in Algorithm 1.

**Algorithm 1** The proposed GAT-LSTM algorithm.**Require:** A set of MIDI data of piano sonatas **Ensure:** Style classification results 1:**procedure** Data Preprocessing 2:   **1.1 Extracting Notes from MIDI Files.** 3:   **1.2 Rhythm Quantization.** 4:   **1.3 Harmonic Analysis.** 5:   **1.4 Constructing the Music Graph.** 6:**end procedure** 7:**procedure** Feature Encoding 8:   **2.1 Node Feature Encoding:** 9:      Use embedding layers to convert categorical features into dense vectors.10:   **2.2 Edge Feature Encoding:**11:      Encode each edge, possibly based on time intervals, harmonic relations, etc.12:      Use different weights to represent different types of edges.13:**end procedure**14:**procedure** GNN and GAT Model Training15:   **3.1 Constructing GNN Layers:**16:      Each GNN layer aggregates the features of the neighbor nodes.17:      Update according to $h_{v}^{\prime }=\mathrm{ReLU}\left(\sum W_{\mathrm{neigh}}h_{\mathrm{neigh}}+W_{\mathrm{self}}h_{v}\right)$.18:   **3.2 Constructing GAT Layers:**19:      Aggregate neighbor node features using attention mechanisms.20:      Attention weight calculation: $\alpha_{uv}=\frac{\exp(\mathrm{LeakyReLU}\left(a^{T}\left[Wh_{u}∥Wh_{v}\right]\right))}{\sum_{k\in N(u)}\exp\left(\mathrm{LeakyReLU}\left(a^{T}\left[Wh_{u}∥Wh_{k}\right]\right)\right)}$21:      Update according to $h_{v}^{\prime }=\sigma \left(\sum_{u\in N(v)}\alpha_{uv}Wh_{u}\right)$.22:   **3.3 Applying LSTM Layers:**23:      Use LSTM layers to capture long-term dependencies.24:      LSTM update rules include gating mechanisms:$$\begin{array}{c} i_{t}=\sigma \left(W_{i}\left[h_{t-1},x_{t}\right]+b_{i}\right),f_{t}=\sigma \left(W_{f}\left[h_{t-1},x_{t}\right]+b_{f}\right),\\ o_{t}=\sigma \left(W_{o}\left[h_{t-1},x_{t}\right]+b_{o}\right),g_{t}=\tanh\left(W_{g}\left[h_{t-1},x_{t}\right]+b_{g}\right),\\ c_{t}=f_{t}⊙c_{t-1}+i_{t}⊙g_{t},h_{t}=o_{t}⊙\tanh\left(c_{t}\right) \end{array}$$25:**end procedure**26:**procedure** Model Training27:   Multi-label Cross-Entropy Loss Function:$$L=-\sum_{i}\sum_{c}\left(y_{ic}\log\left(p_{ic}\right)+\left(1-y_{ic}\right)\log\left(1-p_{ic}\right)\right)$$
where $y_{ic}$ is the real label, $p_{ic}$ is the predicted probability.28:**end procedure**


### 5.3. Details of the Model Training

**Figure 3:**
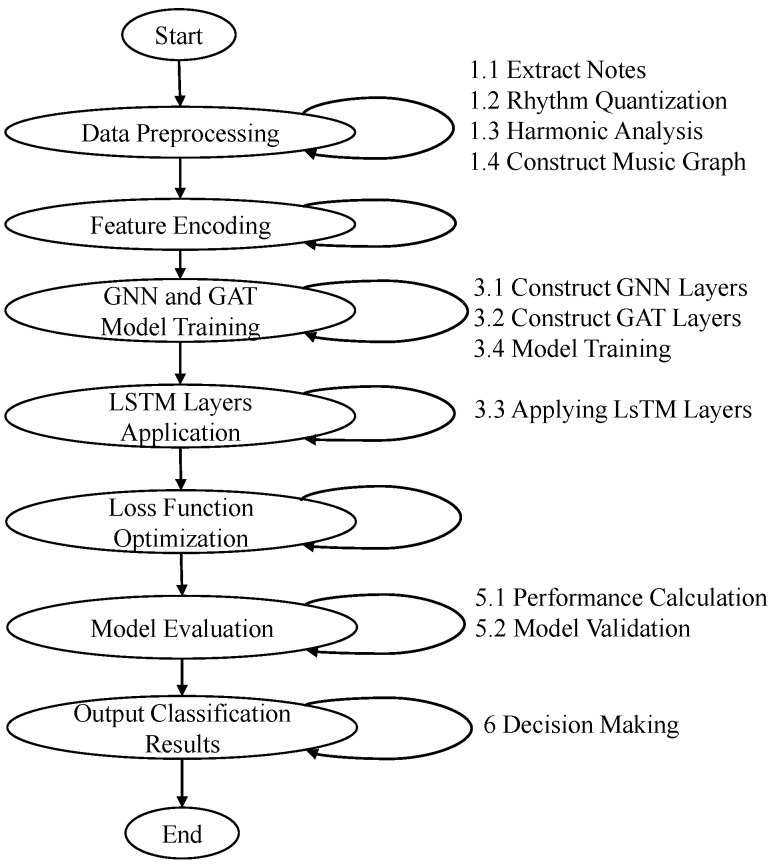
Training procedure for the proposed GAT-LSTM algorithm.

During the model training phase, particular attention is paid to the choice of loss function. The multi-label cross-entropy loss function is chosen because it is suitable for situations in which a piano sonata belongs to multiple emotional and stylistic categories. It considers the interdependence of categories so that the model can perform multi-label classification more accurately. The formula is

(6) $$L=-\sum \sum \left(y_{ic}\log\left(p_{ic}\right)+\left(1-y_{ic}\right)\log\left(1-p_{ic}\right)\right)$$

where $y_{ic}$ is the real label, and $p_{ic}$ is the predicted probability. This loss function can simultaneously handle the presence and absence of each category, and the function calculates the total discrepancies between the predicted probabilities and the actual labels for all of the samples and categories so that the model is more accurate in multi-label classification. Overall, the algorithm proposed here combines the advantages of the GNN and the LSTM to enhance the efficiency and depth of the style and emotion classification of piano sonatas as indicated in Figure 3. Through precise data preprocessing, feature encoding, and efficient model training and evaluation, this algorithm can deeply mine the complex structure and rich emotions in musical works.

In summary, the proposed algorithm integrates the strengths of GNNs and LSTMs, providing a new and effective method for music analysis and offering fresh perspectives to deepen the understanding of the internal structural relationships within musical works. In designing the GAT-LSTM algorithm, we focus on the independent roles of each component as well as their collaboration within the overall framework. Compared with traditional music analysis methods, the GAT-LSTM algorithm is more accurate and delves more deeply into the complex patterns and relationships within musical compositions, thus improving the accuracy and depth of style and emotion classification. The validation of our proposed rational strategies for model training and algorithm refinement is presented in Section 6.

## 6. Experiments and Evaluation

In this section, we describe the training and evaluation of the proposed algorithm, which require the selection of appropriate parameters to optimize the model and ensure good performance on diverse data while preventing overfitting. We introduce the software and hardware conditions needed for the experiments (i.e., the experimental settings) as well as the objectives and evaluation metrics and the analysis of the results.

### 6.1. Experimental Settings

The experiment is designed to identify suitable model parameters that improve the model’s performance and generalization ability and better complete the multi-label classification task. We start with data preprocessing and diversity, adjustment of the hyperparameters and parameters, evaluation indicators, and selection of hardware. First, we divide the piano sonata dataset, which contains a variety of styles and emotions from the Baroque period to the 19th century, into a training set, a validation set, and a test set that serve to adjust the parameters and hyperparameters, prevent overfitting, and evaluate performance of the model, respectively. The simulation of the structure of music generates feature vectors representing pitch, duration, intensity, and so on from the MIDI data so that the model learns and grasps the key features of the sonatas. Second, we choose the appropriate learning rate and batch size to help the model converge to the global optimum faster and improve the generalization performance of the model on unknown data, and, in turn, the classification results. On this basis, we use stochastic gradient descent and Adam to optimize the loss function, maintain the learning rate, and achieve efficient and stable updating of the parameters of the model. Lastly, the experiments are performed on a machine equipped with an Intel Xeon Gold 5222 processor @ 3.80 GHz and 128 GB of RAM. We also set the model evaluation indicators, specifically, standard accuracy, precision, recall, and F1 score, to evaluate the performance of the model comprehensively in various aspects of the music multi-label classification task and also provide a basis for future improvements.

The key experimental parameter settings when designing the learning model for style classification are presented in Table 1.

### 6.2. Construction of the Datasets

Our MIDI dataset was sourced from the Yamaha e-Piano Competition database and the Classical Archives repository, which provide professionally curated MIDI files transcribed from high-quality performances. Each MIDI file underwent rigorous quality checks examining temporal alignment, voice separation, and dynamic accuracy. We verified the accuracy of note events, timing, and articulation against published scores from the Henle and Bärenreiter editions. Files with significant temporal inconsistencies, missing voices, or incorrect note events were excluded. The final dataset includes sonatas evenly distributed across the three periods, with each MIDI file’s encoding quality validated through both automated checks and manual review by professional pianists. Our preprocessing pipeline uses the pretty_midi library to extract note events, maintaining precise onset times, durations, and velocities. We implemented quantization at the 32nd note level to preserve ornamental figures while standardizing rhythmic notation. The resulting representation retains essential performance nuances while ensuring consistent data quality for model training.

Our dataset selection followed a comprehensive framework that ensures balanced representation across historical periods while maintaining rigorous quality standards. We established temporal distribution requirements that capture the evolutionary trajectory of piano sonata composition, mandating equal representation of early, middle, and late works from each period. To avoid the over-representation of any single composer, we implemented a maximum threshold of 30% of works per composer within each period. This balanced approach extends to formal structures, incorporating both conventional and innovative compositional forms that characterize each era. To ensure balanced representation while maintaining dataset diversity, we limited each composer’s contribution to 30% of their total authenticated piano sonata works. For example, Beethoven composed 32 authenticated piano sonatas, so we included 10 sonatas (32×0.3=10) from his repertoire. Similarly, for Mozart’s 18 authenticated piano sonatas, we included 5 sonatas (18×0.3=5). This threshold helps prevent dataset bias toward prolific composers while ensuring sufficient stylistic representation.

The complete dataset comprises 58 piano sonatas: 20 Baroque sonatas (predominantly single-movement works by Scarlatti), 19 Classical sonatas (typically 3–4 movements each), and 19 Romantic sonatas (3–4 movements each). The movement distribution includes 20 single-movement Baroque sonatas, 71 Classical movements, and 100 Romantic movements, totaling 191 individual movements.

The dataset is divided into a training set, a validation set, and a test set at a ratio of 6:2:2. The training set is used to adjust the model parameters and minimize the loss function, thereby improving the accuracy and robustness of the model during the prediction process. The validation sets are used to adjust the model’s hyperparameters, such as learning rate and batch size, etc., and to prevent the model from overfitting. Ultimately, the test set is used to evaluate the model’s performance on unknown data.

### 6.3. Experimental Indicators

In the experimental evaluation of the scheme proposed in this paper, the selection of appropriate evaluation metrics is crucial. We consider classical assessment metrics that have been widely adopted in relevant classification tasks. These metrics not only provide a quantitative measure of the model performance but also ensure the objectivity and comparability of the experimental results. The following key metrics serve in this study to assess the effectiveness of the proposed method.

Accuracy, an essential metric of the performance of the model on the entire dataset, is calculated as

(7) $$\mathrm{Accuracy}=\frac{\mathrm{TP}+\mathrm{TN}}{\mathrm{TP}+\mathrm{TN}+\mathrm{FP}+\mathrm{FN}}$$

Precision is the proportion of instances in which the correctly recognized category is assigned to all assigned categories. High precision indicates a lower rate of false positives, that is, fewer instances in which the style category is incorrectly labeled as true:

(8) $$\mathrm{Precision}=\frac{\mathrm{TP}}{\mathrm{TP}+\mathrm{FP}}$$

Recall is the proportion of genre categories correctly recognized by the model over all true categories. A high recall indicates that the model is able to recognize more real category instances effectively:

(9) $$\mathrm{Recall}=\frac{\mathrm{TP}}{\mathrm{TP}+\mathrm{FN}}$$

The F1 score is the reconciled average of precision and recall, that is, a combination of these two metrics. It is especially important to balance these metrics when dealing with class-imbalanced data:

(10) $$F1\;\mathrm{Score}=2\times \frac{\mathrm{Precision}\times \mathrm{Recall}}{\mathrm{Precision}+\mathrm{Recall}}$$

By comprehensively evaluating the performance of the style classification method proposed in this paper through a consideration of these metrics together, we are able to assess the performance of the various aspects of the proposed style classification method comprehensively. In addition, this multi-perspective evaluation approach can help identify potential improvements in the method so that the performance of the style classification can be further optimized in future work.

### 6.4. Parameter Tuning

In the multi-label classification task, the optimization of the parameters and the selection of hyperparameters have a decisive impact on the performance of the model and the completion of the final classification task. The optimization of the parameters depends on the optimization of the loss function, and the selection of hyperparameters determines the learning ability and generalization ability of the model. Therefore, first, we choose the Adam optimizer based on the gradient optimization algorithm in the model training stage, thereby combining the advantages of the momentum method and RMSProp to ensure fast and stable convergence in the process of finding the optimal loss function. The ADAM optimizer also calculates an independent adaptive learning rate for each parameter and automatically adjusts it based on the update history of the parameter, ultimately affecting the performance of the model and the completion of the classification task. Next, we manually adjust the learning rate based on the parameter update step while avoiding the risk of gradient descent and local optimality and ensuring the fitting ability of the model. We set the learning rate in the range of 0 to 0.1 according to the preliminary experiments. After continuously adjusting the learning rate based on the performance indicators, such as balance recall rate and F1 score, we choose 0.01 as the initial learning rate. This rate is appropriately adjusted in the training to help the model find the global optimal value faster.

Table 2 presents a visualization of the relationship among the parameters, the metrics, and the performance to justify the parameters. We conduct extensive comparisons against state-of-the-art methods in music style classification. The LSTM baseline [11] achieves 70.2% accuracy, demonstrating the limitations of purely sequential modeling. The CNN-LSTM architecture [17] improves performance to 72.5% by incorporating local feature extraction. Sun et al.’s CNN-GAT approach [22] further enhances results to 74.3% through attention mechanisms.

Our integrated GCN-GAT-LSTM model outperforms these baselines by significant margins (3.7–7.8% absolute improvement in accuracy). The performance gain stems from three key factors: (1) effective structural feature extraction through GCN, (2) adaptive feature weighting via the GAT attention mechanism, and (3) temporal dependency modeling with LSTM. The ablation results demonstrate that removing any component reduces performance, confirming each module’s contribution.

The fusion of the GAT and the LSTM is, then, expected to outperform the traditional GAT and GCN methods on all three evaluation metrics. This performance improvement is attributable to the high efficiency of the GAT in capturing the complex relationships between nodes and the power of the LSTM in processing time series data. We expect that the model, by combining these two approaches, will capture the structural features and temporal dynamics of the sonatas more accurately.

**Figure 4:**
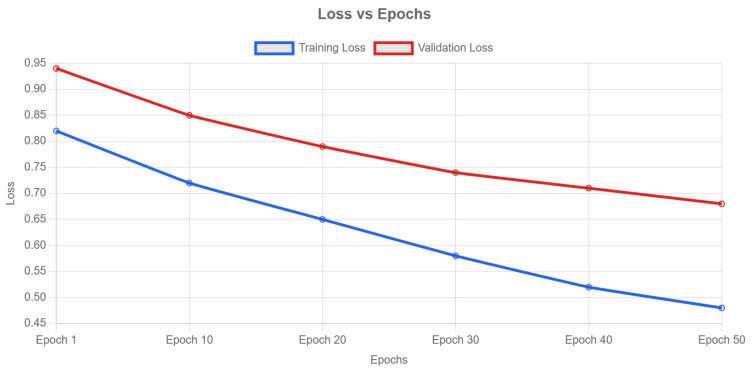
Learning curves of the proposed GAT-LSTM algorithm.

As shown in Figure 4, the training process demonstrates clear learning trends across epochs. The loss curves show consistent convergence, with training loss decreasing from 0.82 to 0.48 and validation loss reducing from 0.94 to 0.68, indicating the model effectively learns both structural and temporal features despite the challenging nature of the multi-style classification task.

We employ early stopping with a patience of 5 epochs to prevent overfitting. The model from epoch 40 is selected for subsequent experiments based on peak validation accuracy (40.2%). While training accuracy continued improving until epoch 50, validation metrics begin declining after epoch 40, indicating potential overfitting. This observation guides our model selection strategy to ensure robust generalization performance.

**Figure 5:**
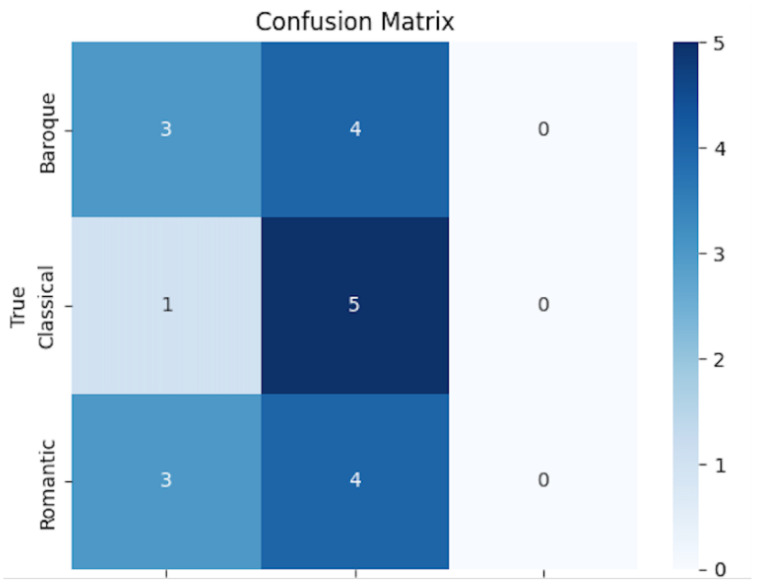
Training process of the proposed GAT-LSTM algorithm.

Figure 5 displays the confusion matrix, which offers deeper insights into the classification results for the three target styles. The matrix reveals that the model performs well in identifying the “Classical” category, with 5 out of 6 instances correctly classified. However, there is some confusion between the “Baroque” and “Romantic” styles, with 3 misclassifications in both directions. This result is consistent with the structural and stylistic similarities shared between these two styles, which can be challenging for any model to distinguish, especially in the context of complex musical compositions like piano sonatas. The confusion between “Baroque” and “Romantic” may be attributed to overlapping harmonic and melodic structures, which the GCN and GAT components of the model analyze. The model performs admirably in extracting key features, but additional fine-tuning, particularly in the attention mechanism or feature extraction steps, could help reduce misclassifications.

### 6.5. Ablation Study

To validate each component’s contribution to the proposed architecture, we conduct comprehensive ablation experiments by systematically removing individual components. As shown in Table 3, removing the GCN component reduces accuracy by 3.8%, demonstrating its crucial role in structural feature extraction. The absence of GAT leads to a 2.9% performance drop, highlighting the importance of attention mechanisms in feature weighting. Most notably, removing LSTM results in a 4.2% decrease in accuracy, confirming the significance of temporal modeling for style classification.

The experiments with paired components reveal that GAT + LSTM achieves better performance (76.3%) compared to GCN + LSTM (75.1%), suggesting the particular effectiveness of attention mechanisms when combined with temporal modeling. However, neither combination matches the full model’s performance, validating our integrated approach. These results empirically demonstrate that each component makes a unique and valuable contribution to the model’s overall effectiveness in piano sonata style classification.

## 7. Conclusions

In this study, we investigate the application of deep learning techniques to classical music analysis, looking specifically at piano sonatas. By combining GNNs with LSTMs, we develop a novel analytical framework that effectively captures the complex structural dynamics inherent in piano sonatas. This approach not only improves the accuracy of style classification but also provides insights into the historical context of these musical works. The evaluations demonstrate that the proposed method efficiently outperforms traditional neural network methods in classifying and analyzing piano sonatas. The model shows a better ability to discriminate subtle musical styles with high precision and recall. In addition, we introduce a comprehensive MIDI dataset of piano sonatas covering various styles and eras, further confirming the utility of the approach and providing a rich resource for current and future research. Future work could involve refining the model to accommodate the diversity of musical expression better and exploring the integration of other modalities, such as audio analysis, to enrich the dataset and depth of analysis.

## Figures and Tables

**Table 1:** Environmental setting.

Environment	Setting
OS	Ubuntu 20.1
Memory	RAM 128 GB
CPU	Intel(R) xx
Learning Framework	Pytorch 1.1.0
Language	Python 3.0.1
Learning rate	0.01
Batch Size	32
Optimizer	Adam

**Table 2:** Model performance.

Method	Accuracy %	F1 Score	Recall Rate
Proposed Method	78.0	77.0	76.0
LSTM [11]	70.2	79.8	68.5
CNN-LSTM [17]	72.5	71.8	70.6
CNN-GAT [22]	74.3	73.5	72.8
GCN	73.0	72.0	71.0
GAT	75.0	74.0	73.0

**Table 3:** Ablation study results.

Model Variant	Accuracy (%)	F1 Score	Recall Rate
Full Model	78.0	77.0	76.0
w/o GAT	75.1	74.3	73.6
w/o LSTM	73.8	72.9	72.1
Only GCN + LSTM	75.1	74.2	73.5
Only GAT + LSTM	76.3	75.4	74.8

## Data Availability

The raw data supporting the conclusions of this article will be made available by the authors on request.

## Nomenclature

$H^{\left(l\right)}$	Node representation of layer *l*
$H^{\left(l+1\right)}$	Updated node representation for layer l+1
$\hat{A}$	Adjacency matrix with added self-connections
$\hat{D}$	Degree matrix corresponding to $\hat{A}$
$W^{\left(l\right)}$	Weight matrix for layer *l*
σ	Activation function (e.g., ReLU)
$h_{i}$	Feature vector of node *i*
$h_{i}^{\prime }$	Updated feature vector of node *i* after GAT layer
$e_{ij}$	Edge between nodes *i* and *j*
$a^{T}$	Parameter vector of the attention mechanism
$\alpha_{ij}$	Attention weight between node *i* and node *j*
*L*	Loss function, typically cross-entropy loss for classification
*N*	Number of samples
*C*	Number of categories
$y_{ic}$	True labels of node *i* using one-hot encoding
$p_{ic}$	Probability of the model predicting that node *i* belongs to category *c*
